# Circulating Irisin Levels Are Positively Associated with Metabolic Risk Factors in Sedentary Subjects

**DOI:** 10.1371/journal.pone.0124100

**Published:** 2015-04-21

**Authors:** María Moreno, José María Moreno-Navarrete, Marta Serrano, Francisco Ortega, Elías Delgado, Cecilia Sanchez-Ragnarsson, Sergio Valdés, Patricia Botas, Wifredo Ricart, José Manuel Fernández-Real

**Affiliations:** 1 Department of Diabetes, Endocrinology and Nutrition, Institut d’Investigació Biomèdica de Girona (IdIBGi), CIBEROBN (CB06/03/010) and Instituto de Salud Carlos III (ISCIII), Girona, Spain; 2 Department of Endocrinology and Nutrition, Hospital Central de Asturias, Oviedo, Spain; 3 Endocrinology and Nutrition, Hospital Regional de Málaga, IBIMA, CIBERDEM, Spain; 4 Department of Medicine, Hospital San Agustin, Aviles, Spain; University of the Balearic Islands, SPAIN

## Abstract

**Introduction:**

A physically active life-style plays an independent role in the protection against type 2 diabetes and cardiovascular diseases. Irisin, a novel exercise-induced myokine, activates thermogenesis in rodents through increasing beige fat cells abundance within white fat. We aimed to investigate circulating irisin levels in association with the degree of physical activity and various metabolic parameters in humans.

**Methods:**

Circulating irisin levels (ELISA) and metabolic parameters were analyzed in 428 subjects (195 men/233 women). Participants were classified according to their self-reported physical activity and to their area of residence.

**Results:**

Circulating irisin levels were higher in active than in sedentary subjects (p= 0.006). Rural inhabitants showed higher circulating irisin levels than urban subjects (p < 0.0001). The increase in irisin levels related to an active lifestyle was only observed in rural citizens (p = 0.014). Among sedentary participants, irisin levels were positively associated with metabolic risk factors (BMI, fasting insulin, HOMA and fasting triglycerides). The area of residence (β= - 0.592, p= < 0.0001) contributed independently to circulating irisin levels variance after controlling for age, gender, BMI, HOMA_IR_, triglycerides and physical activity.

**Conclusions:**

In sedentary participants, circulating irisin levels were positively associated with parameters related to an increased cardiometabolic risk. The present study confirmed that an active lifestyle increases circulating irisin levels, but only among subjects living in a rural environment. Area of residence might be a determinant of irisin levels.

## Introduction

There is accumulating epidemiological evidence that a physically active life-style plays an independent role in the protection against type 2 diabetes, cardiovascular diseases, cancer, dementia and even depression. Current trends confirm that physical activity induces an increase in the systemic levels of a number of cytokines which contribute to tissue communication that is essential to maintain metabolic homeostasis [1]. In this context, the muscle tissue has recently been recognized as an endocrine organ that releases a variety of cytokines, termed myokines, which regulate several physiological and metabolic pathways. Since the discovery of interleukin (IL)-6 as a muscle-secreted cytokine, a number of myokines have been identified, including myostatin, leukemia inhibitory factor (LIF), IL-8 and IL-15 [2, 3].

In 2012, irisin, a new myokine was identified. It is a proteolytic cleavage product of FNDC5 which expression is induced by PPARγ coactivator 1 alpha (PGC1-α). This gene expression is induced by exercise which in turns stimulate irisin releasing into the circulation. This process increase energy expenditure and thermogenesis by increasing uncoupling protein 1 (UCP1) levels via increased PPAR-α expression in white adipose tissue. Irisin is highly conserved among species, with mouse and human being 100% identical, supporting the notion for having a highly conserved function in mammals [4].

Previous reports have shown either mice or human have higher irisin plasma levels after exercise [4, 5]. However, conflicting data on the role of exercise in irisin levels have emerged, since this issue has not been confirmed in a radomized controlled trial performed in 102 untrained participants during 6 months [6] or in an exercise intervention conducted in 16 sedentary overweight/obese subjects during 3 months [7], among others. Indeed, Timmons et al., using gene expression arrays, detected an exercise-induced increase of FNDC5 mRNA in human muscle biopsies from old but not from young subjects [8]. The threshold of exercise required to produce these effects might be crucial, because acute exercise has been demonstrated to result in increased serum irisin concentrations in humans [5, 9]. Recently, Lee et al. have reported that exercise-induced irisin secretion may have evolved from shivering related muscle contraction [10].

Irisin has been proposed to be produced by adipocytes [11, 12], although in lower quantities. Concerning metabolic factors, discrepant results have been obtained in the association between irisin and BMI. While, recent reports have proved that circulating irisin levels were positively correlated with BMI, fasting glucose and total cholesterol [5, 13, 14] other studies found an inverse association with these parameters [12, 15]. In addition, distinct patterns of Fndc5 or irisin have been found in muscle, adipose tissue or in circulation [7]. Metabolic factors such glucose or fatty acids could have a role in irisin regulation [7]. Despite the lack of uniformity, these conflicting results suggested that, body composition, gender, and age may modulate effects of physical exercise on irisin levels.

The stimulation of beige/bride adipocytes in human adipose tissue has been postulated as a possible therapeutic way to improve obesity-associated metabolic disturbances [16]. Since irisin is a circulating factor that activates beige fat cells in rodents [17], it could represent one way to perform this action in humans. The purpose of our study was to investigate the association between an active lifestyle and the circulating irisin levels in a mixed rural—urban Spanish population. To gain insights on the role of irisin in humans, we also examined its associations with various metabolic parameters.

## Methods

### Study population

The Ethical Committee from Investigación del Principado de Asturias affirms that the research Project entitled: "Prevalence of Diabetes Mellitus type II in The Asturian population of 30 years of age or more" has been evaluated and approved by this Committee in October 1997 under number #87/1997. Participants provide their written informed consent to participate in this study. The Asturias Study is a prospective population-based survey of diabetes and cardiovascular risk factors. The baseline examination was carried out during 1998–1999 in the Principality of Asturias in northern Spain. As described in detail previously [18], the population of Asturias is 1 073 761 and mostly Caucasian. Approximately half of the population lives in urban areas. A two-step sampling technique was used. Fifteen basic health areas were selected at random from among the 76 in Asturias with a probability proportional to the number of health cards of users aged between 30 and 75 years. A computer program was then used to randomly select 125 individuals in each basic health area. The final sample size selected was 1 875; 87 individuals were excluded for various reasons (type 1 diabetes, pregnancy, severe disease, hospitalization, or use of diabetogenic drugs). Another 162 individuals were excluded because data needed to contact them were missing. The final sample was composed of 1 626 individuals, of whom 1 034 (63.6%) responded. Vital status and current residency of all individuals were obtained from their health service identification card. Of the original cohort, 42 individuals had died and 19 had left Asturias before the follow-up started. Thirty other individuals were excluded (because of pregnancy, severe disease, hospitalization, or use of diabetogenic drugs). Of the remaining 943 individuals, 700 participated (74.2%). The present study includes only those individuals who did not have diabetes at the baseline.

### Clinical examination

All examinations and analyses were performed at the patients’ local health centers by an endocrinologist and a trained nurse. Information on demographic data and physical activity was obtained by questionnaire used in previous published studies [18]. The participants were asked about the intensity of their leisure time physical activity and whether they practiced any sport. There were three categories: “low,” almost completely inactive; “moderate,” some physical activity 1 h/day, e.g., walking, gardening, dancing, or bicycling; or “high,” vigorous physical activity 3 h/week, e.g., running, swimming, ball games, or competitive sports. The area of residence was classified according to the municipal population: “rural,” < 10 000 inhabitants; “medium,” 10 000–50 000 inhabitants; or “urban,” > 50 000 inhabitants, as described in detail previously [18]. Medical records were reviewed to investigate previous diseases or medication. Height, weight, and BMI (weight in kilograms divided by the square of height in meters) were measured with the subject wearing light clothing and without shoes. Percent fat mass was calculated using the Deurenberg equation [%Fat = 1.20 x BMI + 0.23 x age - 10.8 (if male)- 5.4] [19]. A dichotomous physical activity variable was created based on whether or not the participant met national physical activity guidelines (≥150 minutes/week on ≥ 5 days/week of moderate intensity activity or ≥ 60 minutes/week on ≥ 3 days/week of vigorous intensity activity). Those who met guidelines were classified as active and those who did not were classified as insufficiently active/inactive/sedentary.

### Analytical Methods

The samples were centrifuged in situ using a portable centrifuge. A portable refrigerator containing the samples was taken daily to the Biochemical Laboratory of the Central Hospital of Asturias. Glucose was determined by the hexokinase enzymatic method (Hitachi 747), as described in detail previously [18].

Additional laboratory measurements included total cholesterol, HDL cholesterol, triglycerides (colorimetric method), LDL cholesterol (Friedewald formula), and HbA1c (high-performance liquid chromatography, Adams HA-8160). Serum insulin was measured in duplicate in the same centralized laboratory by a monoclonal immunoradiometric assay (Medgenix Diagnostics, Fleunes, Belgium). The intra-assay CV was 5.2% at a concentration of 10 mU/l and 3.4% at 130 mU/l. The inter-assay CVs were 6.9 and 4.5% at 14 and 89 mU/l, respectively, as described in detail previously [20]. Insulin resistance was determined by the homeostasis model assessment of insulin resistance (HOMA_IR_)

Plasma irisin concentrations were measured within the same day using ELISA (SK00170-01, AVISCERA BIOSCIENCE INC, Santa Clara, California, USA). Intra- and inter-assay coefficients of variation for these determinations were between 4–6% and between 8–10%, respectively.

### Statistics

Statistical analyses were performed using SPSS 19.0 software for Windows (IBM Corp., Armonk, NY, USA). All assays were performed at least in duplicate and reported as mean ± SD. The comparison between groups was performed using two-way ANOVA followed by post-hoc analysis (using DMS and Bonferroni post hoc tests). The relation between variables was analyzed by bivariate correlation (Pearson’s or Spearman’s test) and multiple linear regression models (using stepwise method). Because irisin levels are influenced by the area of residence, the association between irisin and metabolic parameters was calculated by using partial correlation coefficients. Categorical data were expressed as proportion and compared by χ^2^ test. p < 0.05 was considered as statistically significant.

## Results

### Characteristics of the study participants

The clinical and biochemical characteristics of the study subjects are presented in table 1. The range in age was 30.2 to 76.9 years old (median age was 52 years). The range in BMI was 20.6 to 47.8 kg/m^2^. Subjects had a mean plasma irisin concentration of 112.23 ± 66.46 ng/ml, ranging from 31.34 to 336.84 ng/ml.

**Table 1:** Anthropometric and clinical parameters of study subjects according to their area of residence.

	Rural	Medium	Urban
N	123	109	189
Gender (men/women)	57/66	49/60	85/104
Age (years)	53.02 ± 13.37	53.18 ± 12.96	53.55 ± 11.89
SBP (mmHg)	136.60 ± 22.3	135.86 ± 22.33	137.88 ± 21.96
DBP (mmHg)	79.32 ± 10.13	78.01 ± 9.42	79.43 ± 9.01
Weight (kg)	76.79 ± 14.83	74.30 ± 13.63	73.21 ± 12.85
**BMI (kg/m^2^)**	29.29 ± 4.52 ^**#**^	28.37 ± 4.15	27.57 ± 4.23
Percent fat mass (%)	38.4 ± 1 7.6	37.2 ± 8.4	36.2 ± 7.9
Waist circumference (cm)	95.10 ± 13.01	93.13 ± 12.25	92.03 ± 12.68
Hip circumference (cm)	106.49 ± 9.24	105.25 ± 8.5	105.18 ± 8.08
Waist-to-hip ratio	0.89 ± 0.08	0.88 ± 0.07	0.87 ± 0.08
Fasting glucose (mmol/L)	5.50 ± 1.84	5.49 ± 1.18	5.50 ± 1.35
Fasting insulin (pmol/L)	55.08 ± 30.0	51.66 ± 38.09	55.08 ± 31.74
HOMA_IR_	2.32 ± 1.65	2.20 ± 1.92	2.31 ± 1.58
HbA1c (mmol/mol)	30 ± 8.6	30 ± 6.4	29 ± 6.9
Total cholesterol (mmol/L)	5.49 ± 0.94	5.42 ± 0.99	5.56 ± 0.87
**HDL-cholesterol (mmol/L**)	1.41 ± 0.35 ^**#**^	1.48 ± 0.36	1.55 ± 0.41
LDL-cholesterol (mmol/L)	3.45 ± 0.9	3.35 ± 0.87	3.44 ± 0.80
Triglycerides (mmol/L)	1.34 ± 0.72	1.27 ± 0.73	1.24 ± 0.65
Adiponectin (μg/mL)	15.82 ± 8.4	15.73 ± 7.3	14.55 ± 9.7
**Irisin (ng/mL)**	183.8 ± 68.83 * ^**#**^	84.03 ± 32.97	82.11 ± 38.02
**Physical activity (Yes %)**	33.6 *	21.2	23.8

Abbreviations: SBP, systolic blood pressure; DBP, diastolic blood pressure; BMI, body mass index; HOMA, homeostasis model assessment.; HDL, high density lipoprotein; LDL, low density lipoprotein. Mean ± s.d. Statistically significant values are indicated in bold (* = rural Vs medium; # = rural Vs urban).

No significant differences in circulating irisin according to gender were found (114.9 ± 67.9 ng/ml in men vs 108.9 ± 64.6 ng/ml in women, p = 0.3).

### Circulating irisin levels and physical activity

**Fig 1:**
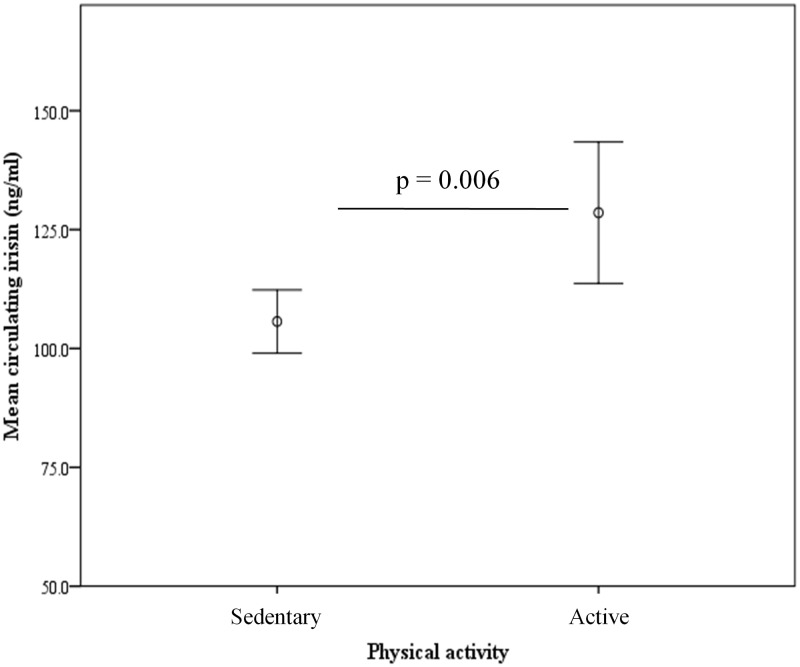
Circulating irisin levels according to self-reported leisure time physical activity in the study participants (n = 428).

A dichotomous physical activity variable was created based on whether (= active subjects) or not (= sedentary subjects) the participants practice any physical activity. Interestingly, circulating irisin levels were significantly higher in active than in sedentary subjects (128.55 ± 78.71 vs 105.66 ± 60.2, p = 0.006) (Fig 1).

Moreover, in order to study the exercise impact on circulating irisin levels in more depth, we have classified subjects into three categories (low, moderate and high), according to their self-reported physical activity. In this regard, no differences were found in circulating irisin levels between participants who displayed low or moderate physical activity (p>0.05). However, subjects with a high physical activity showed higher levels of circulating irisin (128.5 ± 78.7 ng/mL) than participants with a moderate (107.3 ± 64.6 ng/mL, p = 0.021) or low physical activity (102.9 ± 52.8 ng/mL, p = 0.010), respectively.

**Fig 2:**
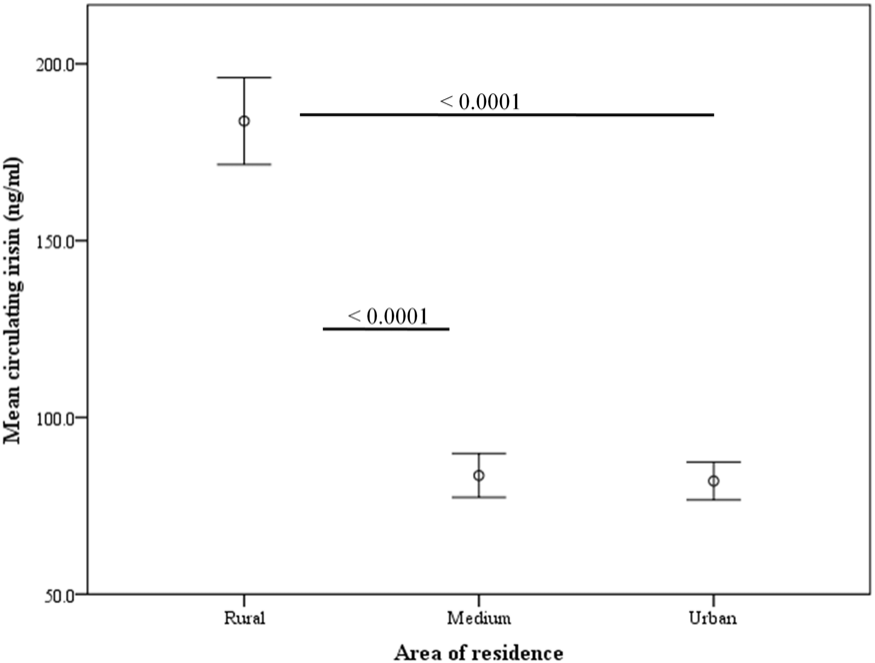
Circulating irisin levels according to the area of residence (n = 428) (only significant differences are shown).

**Fig 3:**
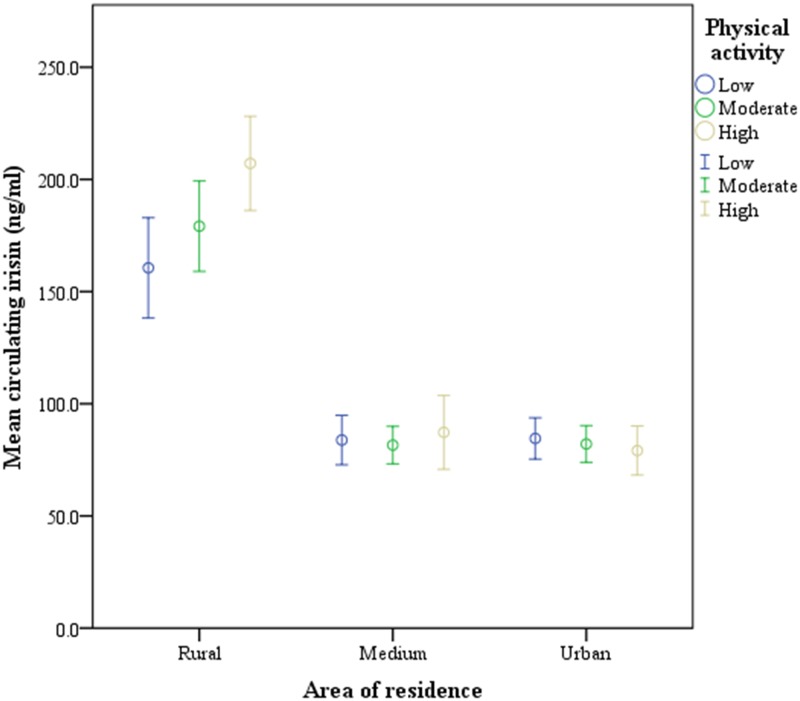
Circulating irisin levels according to the area of residence based on their self-reported leisure time physical activity categories (n = 428) (only significant differences are shown).

Interestingly, by dividing subjects according to their area of residence, rural inhabitants show significantly higher circulating irisin levels than medium or urban subjects (p = < 0.0001). Specifically, in rural inhabitants circulating irisin levels were 183.81 ± 68.8 ng/mL and decreased significatively to 84.03 ± 32.97 and 82.11 ± 38.0 ng/mL in medium and urban subjects, respectively (Fig 2). According to physical activity, we have evaluated the circulating irisin levels in these three different settings, we have found that irisin levels were higher in active than in sedentary subjects only in rural inhabitants (p = 0.014) while no differences were shown in citizens living in urban or medium environments (Fig 3).

### Correlation of circulating irisin with metabolic parameters

In the total study group irisin levels were positively associated to weight (r = 0.129, p = 0.008), BMI (r = 0.148, p = 0.002), HDL (r = -0.194, p = 0.001), triglycerides (r = 0.172, p = <0.0001), insulin (r = 0.113, p = 0.020) and HOMA_IR_ (r = 0.101, p = 0.037). Interestingly, obese subjects (BMI > 30 kg/m^2^) showed higher irisin levels than non-obese participants (BMI < 30 kg/m^2^) (106.93 ± 63.5 vs 124.66 ± 71.5, p = 0.016). Correlation coefficients between irisin levels with the measured clinical parameters according to self-reported leisure time physical activity are presented in Table 2. Among sedentary participants, circulating irisin levels were negatively associated with HDL-cholesterol and positively with BMI, fasting insulin, HOMA_IR_ and total serum triglycerides. Moreover, no significant correlations with age, fasting glucose, HbA1c or adiponectin were found. Surprisingly, in active subjects, a Spearman’s correlation test showed that no parameter was positively associated with circulating irisin levels. However, an inverse association between irisin levels and HDL-cholesterol was observed.

**Table 2:** Bivariate correlation among circulating irisin levels and anthropometric and clinical parameters according to self-reported leisure time physical activity.

	Physical activity			
	Sedentary (n = 317)		Active (n = 110)	
	r	p-value	r	p-value
Age (years)	0.001	0.993	- 0.095	0.325
Weight (kg)	**0.114**	**0.043**	0.158	0.099
BMI (kg/m^2^)	**0.151**	**0.007**	0.170	0.075
Percent fat mass (%)	0.108	0.054	-0.011	0.908
SBP (mmHg)	- 0.036	0.519	- 0.008	0.933
DBP (mmHg)	0.007	0.896	- 0.011	0.908
Waist-to-hip ratio	0.072	0.204	0.149	0.125
Fasting glucose (mg/dL)	0.050	0.375	0.010	0.920
HbA1c (mmol/mol)	0.071	0.207	- 0.029	0.766
Fasting insulin (mU/L)	**0.150**	**0.008**	0.056	0.563
HOMA_IR_	**0.143**	**0.011**	0.037	0.698
Fasting cholesterol (mg/dL)	- 0.014	0.799	- 0.006	0.947
HDL-cholesterol (mg/dL)	**- 0.130**	**0.021**	**- 0.188**	**0.049**
LDL-cholesterol (mg/dL)	- 0.020	0.723	0.038	0.698
Fasting triglycerides (mg/dL)	**0.194**	**0.001**	0.117	0.222

Abbreviations: SBP, systolic blood pressure; DBP, diastolic blood pressure; BMI, body mass index; HOMA, homeostasis model assessment.; HDL, high density lipoprotein; LDL, low density lipoprotein. Statistically significant values are indicated in bold.

In multiple linear regression models, the area of residence (β = - 0.592, p = < 0.0001) contributed independently to circulating irisin levels variance after controlling for age, gender, BMI, HOMA_IR_, triglycerides and physical activity. We also employed general linear model to further address the potential confounding of area of residence in comparison of irisin levels and metabolic parameters. The model revealed that the association between circulating irisin levels and insulin and HOMA_IR_ remained statistically significant after adjustment for the area of residence (p < 0.05).

## Discussion

Similar to the adipose tissue, muscle was also suggested to be an “endocrine organ” with myokines providing cross-talk between adipose tissue, the immune system, hypothalamus and muscle cells [13]. Boström et al. report that exercise training induces FNDC5 gene expression in human muscle, producing irisin, which can convert white fat into brown fat, so enhancing metabolic uncoupling and hence caloric expenditure [4]. In this cross-sectional study, we report the circulating irisin levels in a representative sample of the general population of a whole province in northern Spain, including residents in both urban and rural areas.

A few reports in rodents and humans have shown that exercise increases circulating irisin levels [4, 5] while others have been unable to reproduce the increase in circulating irisin levels after training interventions [6, 7]. Since acute exercise results in increased circulating irisin levels [5, 9], it seems that a threshold of exercise intensity and/or duration is required to produce these effects. Taking all participants as a whole, our analysis derived from adult subjects who regularly practice physical activity reflects that a high level of physical activity is associated with increased circulating irisin levels, at least in a rural environment. Importantly, this was not observed in participants from medium and urban area.

The current study is the first examination of rural-urban differences in circulating irisin levels. We have shown that rural inhabitants display higher circulating irisin levels than urban citizens, both in men and women. For this reason, the influence of rural/urban location prompted us to explore the irisin levels according to the physical activity in each area. Our results show that the increase in circulating irisin levels related to an active lifestyle was only observed in rural citizens and no differences were found in urban or medium inhabitants. However, it has been reported, in diverging populations, the increased cardiometabolic risk in urban populations, suggesting that a good health profile appears significantly more widespread among those living in rural areas [21, 22]. Therefore, besides the augmented cardiovascular risk factors in urban areas, we cannot rule out environmental differences such as food habits or pollution levels between rural and urban settings that might be influencing circulating irisin concentrations. In this regard, persistent organic pollutants have been reported to impair the adipokine axis or fat metabolism, resulting in inappropiated low adiponectin levels [23, 24]. Apart from the theoretical effect of exercise on irisin regulation, other metabolic factors such as glucose or fatty acids have been proposed as important modulators impacting on circulating irisin levels [7]. In this setting, muscle mass was a strong positive predictor of irisin levels, supporting the view that irisin is mainly secreted by the muscle [5, 13]. Anastasilakis et al. have also reported a day-night rhythm of secretion for irisin [9]. Despite the accumulated evidence, the real irisin regulators have not been fully elucidated so far.

By analyzing the association between circulating irisin levels and anthropometric parameters, we have shown a negative association between circulating irisin levels and HDL-cholesterol in both sedentary and active subjects, as recently observed [25]. Moreover, among sedentary subjects, our data reflects that circulating irisin levels were positively associated with parameters related to an increased cardiometabolic risk such as: BMI, fasting insulin, HOMA_IR_ and fasting triglycerides. Previous studies have reported that irisin levels are decreased in patients with type 2 diabetes, while positively correlated with BMI across a broad spectrum of body weights however they have not taken into account the physical activity factor [5, 13, 14]. Because irisin is a myokine that is induced by exercise, it is difficult to evaluate the association between irisin level and clinical parameters without exercise information. In agreement with previous reports [5,16], we hypothesize that the association between irisin levels and metabolic risk factors in sedentary subjects, raises the question of a compensatory role for this myokine to provide a counterbalance to the increased risk of the progression of chronic disorders in an inactive lifestyle. In this sense, Polyzos et al. proposed the term “irisin resistance” or irisin desensitization to explain the apparently discordant upregulation of irisin with obesity and glucose intolerance [26]. Indeed, Kurdiova et al. have suggested that irisin might distinguish different metabolic profiles since distinct patterns of Fndc5 or irisin have been found in muscle, adipose tissue or circulation [7].

With the current study, the circulating irisin level profile has been depicted in a Spanish northern population. We have shown that an active lifestyle increases circulating irisin levels and that irisin is positively associated with several parameters related to an increased cardiometabolic risk, in sedentary subjects. Moreover, we propose that additional factor such as the area of residence might be determinant in the irisin levels. The causality of the observed associations between study parameters should be more intensively studied in prospective cohort and interventional studies. These findings suggest that differences in baseline exercise characteristics between groups should be considered when interpreting results of irisin studies.
